# Birds and Dogs: Toward a Comparative Perspective on Odor Use and Detection

**DOI:** 10.3389/fvets.2018.00188

**Published:** 2018-08-14

**Authors:** Paola A. Prada, Kenneth G. Furton

**Affiliations:** ^1^Department of Environmental Toxicology, Institute for Forensic Science, Texas Tech University, Lubbock, TX, United States; ^2^Department of Chemistry and Biochemistry, International Forensic Research Institute, Florida International University, Miami, FL, United States

**Keywords:** biological detection, odor, canines, birds, olfaction

## Abstract

While canines are generally considered the gold standard for olfactory detection in many situations other animals provide alternatives and offer a unique opportunity to compare biological detection capabilities. Critical components in successfully studying biological detectors is not only understanding their anatomical evidence for olfaction, but also, understanding the life history of the species to better direct the potential of an olfactory task. Here, a brief overview is provided presenting a comparative viewpoint on the use of odors by birds and canines over a range of unique detection scenarios. Similar to canines, birds use olfactory information in various natural oriented contexts where odors are dispersed over a widespread spatial range. Comparing these two distinctive animal models, and current trends in physiological and behavioral assessments may open the door for novel uses of birds as biological sensors in forensic applications.

## Introduction

The term “biological detector” is applied to organismal detectors including animals and plants that can be trained, conditioned or genetically modified to detect key molecules in the environment. The detection of target odor chemicals plays a key role for a variety of purposes within the forensic realm, thus the active research investigating a variety of animal models for the optimal and efficient detection of odors in practical field operations (1–3). With respect to mobile chemical detectors, canines have long been the biological detector of choice, and are currently widely used by law enforcement around the world for detecting a range of forensically important traces. Canines offer clear advantages over instrumental analytical detectors: dogs can easily operate in public; they can be trained to specific odor signatures of target materials, and can track a scent to its source over uneven terrain. These highly mobile biological detectors are also able to pick up and discriminate a specific “scent picture” even against a variety of different “noisy” odor backgrounds. Canine olfaction has been the subject of study from a range of different perspectives. From a physiological standpoint, researchers have been elucidating nasal airflow patterns and their role in odorant transport (4–7). Forensically, canines are one of the most important detection tools for homeland security and law enforcement purposes. Thus, a number of studies have focused on enhancing and understanding canine team performance (8, 9), training regimens (10–12) and clarification of relevant odor chemicals within forensic contexts (13). Clinically, the detection of various types of cancers by canines has been evaluated (14–16). Not surprisingly, the canine olfaction model is widely used when compared to other animal systems. However, it is important to keep in mind that other organisms also use odors in various contexts as observed by their olfactory-related behaviors within their natural environment. Birds are one such animal model that has been largely ignored within an olfaction perspective and more so, in practical, detection capabilities. Birds may represent the next phase in understanding how olfactory cues used across different environmental contexts can prove useful as a biological detection model if directed toward more focused olfactory detection tasks. This paper outlines avian olfaction evidence presenting three bird species as models (i.e., homing pigeons, turkey vultures and domestic chickens) and highlights how birds' intrinsic life history olfactory traits, even though greatly overlooked for biological detection, can be potentially directed to similar detection tasks as that observed in canine forensic field-based operations.

## What are some uses of odors in avian species?

Both canines and birds use olfactory evidence over a range of unique detection viewpoints. However, as opposed to canines, avian olfactory capabilities have been substantially overlooked by a historical belief that birds are anosmic (i.e., having little or no smell) (17). However, over the past 50 years, researchers have shown the use of olfaction by birds in a range of biological contexts ranging from navigation and foraging to species, sex, and individual odor recognition (18–23). Since the seminal work of Bang in 1960, the anatomical evidence for avian olfaction surfaced in the scholarly literature (17). As part of this morphological evidence in the olfactory functioning of birds, continuous research focused on comparing olfactory bulbs across species (24). This early survey suggested that kiwis, tube-nosed marine birds and some vulture species, had among the largest olfactory bulbs. However, the relative importance of this morphological value with the olfaction modality in avian species was not fully understood and subsequently has become an area of fruitful biological research (See Table 1) (23). Olfactory-driven behaviors in birds can be discussed in relation to specific natural contexts and for purposes of this paper, the bridge between these natural traits will be linked to their potential forensic approaches. A description of three avian model systems will be presented: homing pigeons, turkey vultures, and domestic chickens.

**Table 1:** Comparison of olfactory bulb to brain ratios, adapted from Bang and Cobb 1968.

**ORDER/Species**	**Olfactory Bulb Diameter (mm)**	**Cerebral Hemisphere (mm)**	**Bulb/Hemisphere Ratio**
**APTERYGIFORMES**			
Kiwi *Apteryx Australia*	12.0	35.0	34.0
**PROCELLARIIFORMES**			
Snow Petrel *Pagodroma nivea*	6.7	18.0	37.0
Wilson's petrel *Oceanites oceanicus*	3.6	10.8	33.0
Wedge-tailed Shearwater *Puffinus pacificus*	5.5	17.8	30.0
Greater Shearwater *Puffinus gravis*	6.0	20.0	30.0
Dove Prion *Pachyptila desolata*	4.1	14.0	29.5
Black-footed Albatross *Diomedea nigripes*	8.0	28.0	29.0
California Shearwater *Puffinus opisthomelas*	5.0	17.0	29.0
Cape Pigeon *Daption capensis*	5.5	20.0	27.5
Fulmar *Fulmarus glacialis*	5.7	21.0	27.0
Diving Petrel *Pelecanoides georgicus*	2.0	11.3	18.0
**COLUMBIFORMES**			
Wild Blue Rock Pigeon *Columba livia*	2.9	13.7	22.0
Domestic Rock Pigeon *Columba livia*	2.0	11.0	18.0
**FALCONIFORMES**			
Turkey Vulture *Cathartes aura*	6.0	24.0	28.7
**GALLIFORMES**			
Domestic Fowl *Gallus gallus*	2.0	13.0	15.0

### Homing pigeons

**Figure 1:**
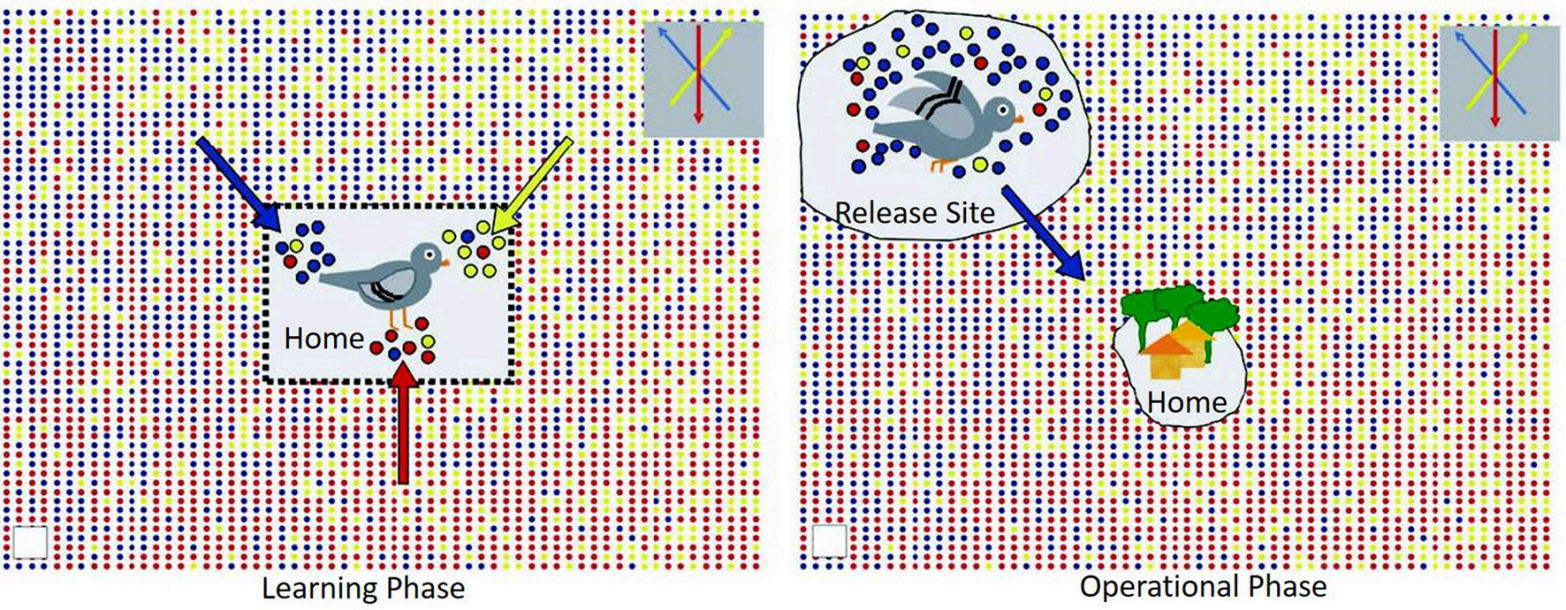
Graphical representation of the environmental odor blend hypothesis in pigeon navigation; with permission Gagliardo (27).

One of the most common avian models to study animal navigation has been the domesticated rock pigeon (*Columba livia*). Beyond their fascinating natural traits, the homing pigeon has been used in a variety of field operations including to transport messages and carry small light-weight packages, including smuggling contraband into prisons or carry messages in times of war/conflict. Undoubtedly their level of intelligence cannot be ignored, but more so, is understanding their ability to travel hundreds of kilometers to and from their home loft even after being released in completely unfamiliar territory. This is where the concept of olfactory navigation behavior is important for these types of potential applications. Back in the early 1970s, Papi et al. conducted pioneering studies with a group of pigeons with their olfactory nerves sectioned and an intact control group of pigeons (25). Only the intact group returned to the home loft. Furthermore, at around the same time, another experiment conducted by Wallraff introduced the hypothesis that indeed these birds used an environmental odor picture directly linked to their successful navigation back home (26). The idea behind an environmental odor picture is that pigeons are able to learn and associate these environmental “local” odors in conjunction with other factors such as wind. Hence, when left in unfamiliar territory, they are able to identify this odor “bouquet” and remember the direction and displacement of these environmental olfactory cues eventually leading them back home (Figure 1) [see review,(27, 28)]. This seminal olfactory navigation model basically branches into two distinctive steps, the first one where the pigeons learn the wind-borne odors in their home loft surroundings along with the wind direction (29) and secondly, an active operational step where the relocated bird can determine direction of displacement by identifying local odors and remembering where these local odors came from at the home loft (27, 30). The definition of this environmental odor blend was furthered validated by a model suggesting an explicit spatial “network” of odor gradients which is directly linked to location estimation relative to the loft. In this work, instrumental analysis of air samples was studied at 96 sites over a radius of 200 km showing that indeed there is a rather stable gradient ratio of hydrocarbons that interact with wind patterns which birds could utilize for navigation (31). To this day, this “volatile atmospheric odor picture” is the subject of active research not only in pigeons (32) but extending the experimental approach to other wild birds (33). Thus, active experimental evidence seems to highlight the capability of the homing pigeon to sample their local odor gradients as a mechanism to establish navigation. Even though the actual compounds used for this purpose is still an area not yet fully understood, the potential for directing pigeons toward specific target odor chemicals by a constant exposure in their home loft environment could be an olfactory task developed for focused detection missions.

### Turkey vultures

As observed from Table 1, the turkey vulture has one of the largest olfactory bulbs of any bird (23). These birds are normally associated with their rapid presence at scenes with decomposing tissue and dead animals. Hence, it is not unexpected that this avian specie has been the subject of study in numerous forensic taphonomic experiments. But before taking a forensic perspective, it is important to understand their olfactory tracking capabilities. Stager (34) conducted detailed field experiments where he noted that odors from both fresh and decomposing animal tissue, produced positive olfactory responses from turkey vultures. In his pioneering work, he also noted that this avian specie was able to detect the presence of hidden animal baits thus further strengthening the olfaction modality used in food location. Stager further describes how turkey vultures were attracted to a volatile organosulfur compound, ethyl mercaptan, used by oil company engineers as an odorant for gas leak detection (35). Hence, foundational observations led to the suggestion that olfaction indeed played a significant role in the life history of this bird. Other studies have even suggested that turkey vultures can discern the age of the carcass. Houston (36) performed experiments where turkey vultures were efficient at locating 1-day old carcasses while rejecting completely rotten meat. Thus, olfaction in this animal model can play a significant role for food location and also highlights a distinctive odor picture of the condition of their prey.

From a forensic viewpoint, vulture species have been the subject of study in terms of the effects of scavenging on human remains. For forensic investigators, animal scavenging can disrupt the crime scene by the dispersal of remains far from the location of interest and also represents a challenge in the estimation of the postmortem interval (PMI). However, regardless of the problems faced by crime scene investigation (CSI) teams by the effect of these scavenging activities, this avian model showcases a keen sense of olfaction for the decomposition odor plume. Reeves (37) used pig carcasses as scavenging targets in the central Texas region during summer months. Both black and turkey vultures waited 24 h before scavenging activities and skeletonized the carcasses in as fast as 3 h. Compared to this study, another experiment conducted in Southern Illinois highlighted that there was a delay in the time of first vulture arrival (up to 28 days), much slower feeding times on the pig remains. Hence, this study suggests an effect that vulture scavenging is directly linked to geographical region and climate (38). Furthermore, using spatial analytical methods, researchers have observed how skeletal remains are dispersed by vultures to lower elevations, and that such dismemberment and dispersal occurs during early phases of the scavenging activity (39). Even though olfactory studies in this avian species are limited, there is evidence of their olfactory detection toward a “decomposition odor blend” that has direct practical implications for future research in the area in terms of decomposition odor stages (as that seen with the condition of their prey) and in the potential identification of decomposing human remains.

### Domestic chicken

Like pigeons, the domestic chicken is a familiar avian model within various biological experimental contexts. In a review by Jones and Roper (40), the functional significance of an olfactory modality is described in the domestic fowl. This animal system has been studied with respect to odorant exposure in terms of their rearing environment and chemosensory learning aspects. Studies with odorants such as isoamyl acetate, eugenol, and allyl sulfide demonstrated that 1-day old chicks showed differential sensitivity to different odorants at varying concentrations (41). Furthermore, evaluation of odorant exposure to chicks pre-hatching has also been investigated with stimulus such as strawberry demonstrating that a chick's chemosensory preferences are changed with a pre-hatching exposure to the desired stimulus thereby implying olfactory learning (42). Variations of these early exposure experiments have included a range of odorants (see review by Jones and Roper) (39), and also a variety of different methods of odor presentation (43–45). In other behavioral assessments, fecal predator odor was presented to domesticated chickens and showed that individuals can respond to predator olfactory cues, as observed by their decreased foraging and increased vigilance, without any prior odorant exposure or learning (46). Furthermore, it has been shown that even a blend of odorants representing a “motherly” scent reduces stress as determined by a range of physical and behavioral parameters (47). In a study conducted by Bertin et al. (48), a pre-hatch effect of the intensity of odor signals in the regulation of later feeding behavior was reported, thereby highlighting the capability of embryo chemosensory learning. Collectively, these findings provide growing support for the role of olfactory cues in this avian species in a series of chemical communication purposes.

## How can avian use of odors be compared to canine biological detection?

The presented avian models emphasize the distinctive use of odors in their natural contexts. From navigation, food searching, to olfactory learning, these three presented avian species corroborate the use of olfaction. However, what has yet to be exploited is the potential uses of these natural olfactory-mediated behaviors in a more practical biological detection context, namely forensic detection and a direct comparison to the canine model. As stated previously, canines are the biological detector of choice, specifically in the realm of law enforcement and security purposes. In terms of biosensor applications, other animal models such as rats, bees, wasps (49), and elephants (50) have demonstrated such potential applications. To date, the avian species has not been the focus of any study for forensic odorant detection applications.

One reason for the neglect of avian biological detection could be that olfaction is not historically been considered a major sensory modality in birds. Perhaps, a general lack of recognition of the importance of olfaction in birds has misguided our efforts, despite the evidence, that avian species could be redirected for detection roles. When looking at the 3 avian models presented in this paper, olfaction in birds plays a key role for chemical signaling, communication, odor learning and exposure, early animal experience, and as a housing or environmental enrichment (as seen with navigation). The environmental enrichment can be observed by the ability of birds (as seen with the pigeon model) to learn environmental odors in association with wind direction, which highlights how the environment provides an odor source they are able to recognize to determine displacement direction (26). All of these olfactory-guided contexts are shared by the canine species. The only difference between these 2 species is the application to practical forensic detection roles.

### The environmental odor bouquet of birds and dogs

Using the “odor map” model of avian olfaction as that observed with pigeons for navigation, a comparative viewpoint can be made with the odor plume encountered by a canine during their search pattern behavior. This spatial odor gradient map suggested by Wallraff (25, 30, 51) in terms of sampling the air to obtain environmental odor cues for directionality, can be directly linked to canine's directional tracking. Whether it be for operational tasks such as finding a missing person or in search and rescue missions, the success of this olfactory role is in the canine's ability to sample the surrounding air for directionality as to the whereabouts of the target's location. In this case, the canine is not finding home (as the pigeon with the home loft) but his trained target odor. Studies in canine olfaction have embarked on evaluating the behavior of dogs during this olfactory tracking. Thesen et al. (52) evaluated 4 trained German shepherd tracking dogs using 20-min old tracks on grass and 3-min old tracks on concrete. They recognized three distinctive phases, an initial searching phase, a deciding phase (determination of directionality) and a tracking phase. Thus, the study demonstrated the need of the canine to obtain olfactory cues from the environment and points to the sensitivity in detecting specific substances for successful tracking. Other studies have even compared olfactory and visual cues for directionality of tracking (53) and also evaluated the amount of discrete information needed (5 sequential footsteps) to determine the direction of an odor trail (54). Thus, this tracking example within the canine model can be directly compared to the pigeon navigation model where the bird “samples the air” to determine directionality in relation to the home loft. The notion of an environmental odor blend capable of yielding these olfactory cues for navigation/tracking purposes exists for both species, thus the possibility of directing this navigation olfactory behavior (as in the case of birds) to specific chemical odor blends of forensic importance, within an avian species, is certainly not so implausible.

### A decomposition outlook

A growing area of operational use and research needed within canine biological detection has been that observed in the detection of human remains, or the use of the so-called cadaver dog. Not only for particular crime scene processing issues (i.e., clandestine graves) but also in contexts such as those observed after natural disasters where these canine teams are deployed to help locate and identify deceased victims under difficult terrains and rubble piles. Many research groups have focused on understanding the chemical odor blend of decompositional odor to gain a perspective on this volatile odor profile. Vass et al. (55–57) initiated the establishment of an odor database of human remains showing chemical trends for volatile organic compounds detected utilizing triple sorbent traps. The understanding of a human decomposition odor picture plays a key part in better directing optimal training procedures for biological detection. Hence, other work in the area has focused on the development and analysis of synthetic training aids (58–60), influence of age, soil textures, and surfaces on chemical odor profiles (61–63), to name a few. Recently, the introduction of 2-dimensional gas chromatography and time of flight mass spectrometry has been the subject of intensive decomposition odor profiling studies (64, 65). Having a context of the vast amount of research in the area of human decomposition odors, only calls for alternate pathways of detection. As observed in the above discussion on vulture scavenging, and their natural olfactory behavior in finding their decomposing prey, this avian specie could represent an experimental model to further investigate target compounds of interest within a decomposition odor picture. From the knowledge gained from decompositional odor databases using instrumental analysis, researchers can target compound validation using avian models such as turkey vultures to verify odor mixtures and concentration thresholds that yield positive avian response. Different odor blends can be prepared and demonstrate attraction or aversion to better understand human decomposition using an altogether novel biological system.

### A forensic perspective on odor exposure

Under operational conditions, canine biological detection revolves around routine maintenance training of the target odor(s) for the corresponding mission. Hence, it is imperative that optimal performance be assessed not only on the behavioral aspect, but also in a thorough understanding of the target odor chemicals involved in that positive alert. Advances in the analytical forensic laboratory have resulted in increasingly lower detection thresholds allowing the elucidation of some of these volatile odor chemical signatures (13). Explored forensic areas have included narcotics (66–68), explosives (69, 70), and human scent (71). Regardless of the specific area of detection, a common factor of study is the underlying link between odor exposure and the behavior of the canine with respect to that odor mixture. Different studies have geared to understand effects of extraneous odors on canine detection sensitivity (72), while others have tested the number of substances trained with respect to detection performance (12). Just as that seen with the decomposition odorous blend, a key aspect in optimal detection is the ability to provide efficient training aids. Thus, comparing this need and area of research, a comparative perspective can be bridged with the domestic chicken as an avian model. Odor exposure pre-hatching and the link to odor learning can be extended to employing odor chemical of forensic importance in order to establish baseline odor thresholds within an avian species under controlled laboratory conditions. Comparing to practical canine models of experimentation, where different training aids, and/or target odor chemical are presented for behavioral responses, the domestic chicken can be trained to detect similar odor chemicals, developing the possibility of detection by indicating presence/absence of stimuli in natural environments. Initial work in this area has demonstrated that birds can be trained using the same forensic target odors used by dogs (73).

## Conclusion

The role of biological detection in the area of forensic science and national security has witnessed an increase of active research investigating various animal models, with canines, being the most optimized and best-known working animal model to date. However, animal systems such as birds provide an olfactory foundational framework worth exploring further, and which to date, has largely been ignored within a practical operational perspective. It is important to inform how scientific support highlights an active and varied role of different olfactory-mediated behaviors within the avian species. Canine detection science has become a key tool in many forensic applications, but by working on a parallel level, birds can represent an avenue of olfactory detection that provides yet another pathway for implementation. It is critical to the success of biological detection to assess alternate models that represent solid evidence-based characteristics of fine-tuned olfactory capabilities. This review serves as a call for the research community to consider a different perspective on birds as a viable and working system for comparative olfactory tasks as that observed with working dogs simply taking into consideration already existing life history olfactory traits.

## Author contributions

PP contributed with overall writing and preparation of the article, appropriate literature review and general outline of review. KF contributed with general outline of review, presentation of topic, editing and review.

### Conflict of interest statement

The authors declare that the research was conducted in the absence of any commercial or financial relationships that could be construed as a potential conflict of interest.

## References

[1] Habib MK. Controlled biological and biomimetic systems for landmine detection. Biosens. Bioelectron. (2007) 23:1–18. 10.1016/j.bios.2007.05.005 17662594

[2] Leitch O Anderson A Kirkbride KP Lennard C. Biological organisms as volatile compound detectors. Forensic Sci. Int. (2013) 232:92–103. 10.1016/j.forsciint.2013.07.004 24053870

[3] Oh Y Lee Y Heath J Kim M Applications of animal biosensors: a review. IEEE Sens. J. (2015) 15:637–45. 10.1109/JSEN.2014.2358261

[4] Craven BA Neuberger T Paterson EG Webb AG Josephson EM Morrison EE. Reconstruction and morphometric analysis of the nasal airway of the dog (*Canis familiaris*) and implications regarding olfactory airflow. Anat. Rec. (2007) 290:1325–0. 10.1002/ar.20592 17929289

[5] Craven BA Paterson EG Settles GS. The fluid dynamics of canine olfaction: unique nasal airflow patterns as an explanation of macrosmia. J. R. Soc. Interface (2010) 7:933–43. 10.1098/rsif.2009.0490 20007171 PMC2871809

[6] Lawson MJ Craven BA Paterson EG. A computational study of odorant transport and deposition in the canine nasal cavity: implications for olfaction. Chem. Senses. (2012) 37: 553–566. 22473924 10.1093/chemse/bjs039

[7] Settles GS Kester DA Dodson-Dreibelbis LJ The external aerodynamics of canine olfaction, in: Barth FG J. Humphrey AC Secomb TW editors. Sensors and Sensing in Biology and Engineering. Vienna; New York, NY: Springer (2002), p. 323–35.

[8] Cobb M Branson N McGreevy P Lill A Bennett P. The advent of canine performance science: offering a sustainable future for working dogs. Behav. Process. (2015) 110:96–104. 10.1016/j.beproc.2014.10.012 25444772

[9] Porritt F Shapiro M Waggoner P Mitchell E Thomson T Kacelnik A Performance decline by search dogs in repetitive tasks, and mitigation strategies. Appl. Anim. Behav. Sci. (2015) 166:112–22. 10.1016/j.applanim.2015.02.013

[10] Porritt F Mansson R Berry A Cook N Sibbald N Nicklin S Validation of a short odour discrimination test for working dogs. Appl Anim Behav Sci. (2015) 165:133–42. 10.1016/j.applanim.2014.11.021

[11] Meyer Ladewig J The relationship between number of training sessions per week and learning in dogs. Appl Anim Behav Sci. (2008) 111:311–20. 10.1016/j.applanim.2007.06.016

[12] Williams M Johnston JM Training and maintaining the performance of dogs (*Canis familiaris*) on an increasing number of odor discriminations in a controlled setting. Appl. Anim. Behav. Sci. (2002) 78:55–65. 10.1016/S0168-1591(02)00081-3

[13] Furton KG Caraballo NI Cerreta MM Holness HK. Advances in the use of odour as forensic evidence through optimizing and standardizing instruments and canines. Phil Trans R Soc B Biol Sci. (2015) 370:20140262. 10.1098/rstb.2014.0262 26101287 PMC4581006

[14] Hackner K Errhalt P Mueller MR Speiser M Marzluf BA Schulheim A. Canine scent detection for the diagnosis of lung cancer in a screening-like situation. J Breath Res. (2016) 10:046003. 10.1088/1752-7155/10/4/046003 27677188

[15] Jezierski T Walcsak M Ligor T Rudnicka J Buszewski B. Study of the art: canine olfaction used for cancer detection on the basis of breath odour. Perspectives and limitations. J. Breath Res. (2015) 9:027001. 10.1088/1752-7155/9/2/027001 25944810

[16] Moser E McCulloch M Canine scent detection of human cancers: a review of methods and accuracy. J Vet Behav. (2010) 5:145–52. 10.1016/j.jveb.2010.01.002

[17] Nevitt GA Prada PA The chemistry of avian odors: an introduction to best practices. In: Richard Doty L editor. Handbook of Olfaction and Gustation, 3rd Edn. New York, NY: Wiley-Liss (2015), p. 565–77.

[18] Bang BG. Anatomical evidence for olfactory function in some species of birds. Nature (1960) 188:547–9. 13686568 10.1038/188547a0

[19] Caro SP Balthazart J Bonadonna F. The perfume of reproduction in birds: chemosignaling in avian social life. Horm Behav. (2015) 68:25–42. 10.1016/j.yhbeh.2014.06.001 24928570 PMC4263688

[20] Roper TJ Olfaction in birds. Adv Study Behav. (1999) 28:247–332.

[21] Stager KE Avian olfaction. Am Zool. (1967) 7:415–9.

[22] Wallraff HG Avian olfactory navigation: its empirical foundation and conceptual state. Anim. Behav. (2004) 67:189–204. 10.1016/j.anbehav.2003.06.007

[23] Wenzel BM Avian olfaction: then and now. J Ornithol. (2007) 148:S191–4. 10.1007/s10336-007-0147-z

[24] Bang BG Cobb S The size of the olfactory bulb in 108 species of birds. Auk (1968) 85:55–61.

[25] Papi F Fiore L Fiaschi V Benvenuti S The influence of olfactory nerve section on the homing capacity of carrier pigeons. Monit Zool Ital. (1971) 5:265–7.

[26] Wallraff HG Weitere Volierenversuche mit Brieftauben: wahrscheinlicher Einfluss dynamischer Faktorender Atmosphare auf die Orientierung. Z. Vgl. Physiol. (1970) 68:182–201.

[27] Gagliardo A. Forty years of olfactory navigation in birds. J Exp. Biol. (2013) 216:2165–71. 10.1242/jeb.070250 23720797

[28] Wallraff HG. Simulated navigation based on observed gradients of atmospheric trace gases (Models on pigeon homing, Part 3). J Theor Biol. (2000) 205:133–45. 10.1006/jtbi.2000.2052 10860706

[29] Ioalè P Nozzolini M Papi F Homing pigeons do extract directional information from olfactory stimuli. Behav. Ecol. Sociobiol. (1990) 26:301–5.

[30] Papi F Fiaschi V Benvenuti S Baldaccini E Pigeon homing: outward journey detours influence the initial orientation. Monit. Zool. Ital. (1973) 7:129–33.

[31] Wallraff HG Andreae MO Spatial gradients in ratios of atmospheric trace gases: a study stimulated by experiments on bird navigation. Tellus (2000) 52B:1138–57. 10.3402/tellusb.v52i4.17084

[32] Gagliardo Pollonara E Wikelski M Pigeon navigation: exposure to environmental odours prior release is sufficient for homeward orientation, but not for homing. J. Exp. Biol. (2016) 219:2475–80. 10.1242/jeb.140889 27284069

[33] Safi K Gagliardo A Wikelski M Kranstauber B. How displaced migratory birds could use volatile atmospheric compounds to find their migratory corridor: a test using a particle dispersion model. Front Behav Neurosci. (2016) 10:175 10.3389/fnbeh.2016.00175 27799899 PMC5065961

[34] Stager KE The role of olfaction in food location by the turkey vulture (*Cathartes aura*). Contrib Sci. (1964) 81:1–63.

[35] Wetmore The role of olfaction in food location by the turkey vulture (*Cathartes aura*) by Kenneth Stager. Auk. (1965) 82:661–2.

[36] Houston DC Scavenging efficiency of turkey vultures in tropical forest. Condor (1986) 88: 318–23.

[37] Reeves NM. Taphonomic effects of vulture scavenging. J Forensic Sci. (2009) 54:523–8. 10.1111/j.1556-4029.2009.01020.x 19432736

[38] Dabbs GR Martin DC. Geographic variation in the taphonomic effect of vulture scavenging: the case for southern Illinois. Forensic Sci J. (2013) 58:S20–5. 10.1111/1556-4029.12025 23181511

[39] Spradley MK Hamilton MD Giordano A. Spatial patterning of vulture scavenged human remains. Forensic Sci Int. (2012) 219:57–63. 10.1016/j.forsciint.2011.11.030 22204892

[40] Jones RB Roper TJ Olfaction in the domestic fowl: a critical review. Physiol Behav. (1997) 62:1009–18. 9333194 10.1016/s0031-9384(97)00207-2

[41] T.Burne HJ Rogers LJ Responses to odorants by the domestic chick. Physiol Behav. (1996) 60:1441–7. 8946488 10.1016/s0031-9384(96)00300-9

[42] Sneddon H Hadden R Hepper PG. Chemosensory learning in the chicken embryo. Physiol Behav. (1998) 64:133–9. 9662076 10.1016/s0031-9384(98)00037-7

[43] Jones RB Carmichael NL Domestic chicks are attracted to a familiar odorant in a novel test situation: a brief report. Appl Anim Behav. Sci. (1999) 61:351–6.

[44] Burne TH Rogers LJ Changes in olfactory responsiveness by the domestic chick after early exposure to odorants. Anim Behav. (1999) S8:329–36. 10.1006/anbe.1999.1151 10458884

[45] Porter RH Hepper PG Bouchot C Picard MA. simple method for testing odor detection and discrimination in chicks. Physiol Behav. (1999) 67:459–62. 10497967 10.1016/s0031-9384(99)00056-6

[46] Zidar J Lovlie H Scent of the enemy: behavioural responses to predator faecal odour in the fowl. Anim Behav. (2012) 84:547–54. 10.1016/j.anbehav.2012.06.006

[47] Madec I Gabarrou JF Saffray D Pageat P Broilers (*Gallus gallus*) are less stressed if they can smell a mother odorant. S Afr J Anim Sci. (2008) 38:201–6. 10.4314/sajas.v38i3.4126

[48] Bertin A Calandreau L Arnould C Nowak R Levy F In ovo olfactory experience influences post-hatch feeding behaviour in young chickens. Ethology (2010) 116:1027–37. 10.1111/j.1439-0310.2010.01820.x

[49] Frederickx Verheggen FJ Haubruge E Biosensors in forensic sciences. Biotechnol Agron Soc Environ. (2011) 15:449–58.

[50] Miller AK Hensman MC Hensman S Schultz K Reid P Shore M African elephants (*Loxodonta africana*) can detect TNT using olfaction: implications for biosensor application. Appl Anim Behav Sci. (2015) 171:177–83. 10.1016/j.applanim.2015.08.003

[51] Wallraff HG Navigation by homing pigeons. Ethol Ecol Evol. (1990) 2:81–115.

[52] Thesen Steen JB Doving KB. Behaviour of dogs during olfactory tracking. J Exp Biol. (1993) 180:247–51. 8371085 10.1242/jeb.180.1.247

[53] Wells DL Hepper PG Directional tracking in the domestic dog, *Canis familiaris*. Appl Anim Behav Sci. (2003) 84:297–305. 10.1016/j.applanim.2003.08.009

[54] Hepper PG Wells DL. How many footsteps do dogs need to determine the direction of an odour trail? Chem Senses. (2005) 30:291–8. 10.1093/chemse/bji023 15741595

[55] Vass AA Smith RR Thompson CV Burnett MN Wolf DA Synstelien JA. Decompositional odor analysis database. J Forensic Sci. (2004) 49:760–9. 10.1520/JFS2003434 15317191

[56] Vass AA Smith RR Thompson CV Burnett MN Dulgerian N Eckenrode BA. Odor analysis of decomposing buried human remains. J Forensic Sci. (2008) 53:384–91. 10.1111/j.1556-4029.2008.00680.x 18366571

[57] Vass AA. Odor mortis. Forensic Sci Int. (2012) 222:234–41. 10.1016/j.forsciint.2012.06.006 22727573

[58] DeGreeff LE Weakley-Jones B Furton KG. Creation of training aids for human remains detection canines utilizing a non-contact, dynamic airflow volatile concentration technique. Forensic Sci Int. (2012) 217:32–8. 10.1016/j.forsciint.2011.09.023 22018852

[59] Stadler S Stefanuto PH Byer JD Brokl M Forbes S Focant JF. Analysis of synthetic canine training aids by comprehensive two-dimensional gas chromatography-time of flight mass spectrometry. J Chromatogr A. (2012) 1255:202–6. 10.1016/j.chroma.2012.04.001 22554412

[60] Tipple CA Caldwell PT Kile BM Beussman DJ Rushing B Mitchell NJ. Comprehensive characterization of commercially available canine training aids. Forensic Sci Int. (2014) 242:242–54. 10.1016/j.forsciint.2014.06.033 25093917

[61] Alexander MB Hodges TK Bytheway J Aitkenhead-Peterson JA. Application of soil in forensic science: residual odor and HRD dogs. Forensic Sci Int. (2015) 249:304–13. 10.1016/j.forsciint.2015.01.025 25747330

[62] Alexander MB Hodges TK Wescott DJ Aitkenhead-Peterson JA. The effects of soil texture on the ability of human remains detection dogs to detect buried human remains. J Forensic Sci. (2016) 61:649–655. 10.1111/1556-4029.13084 27122400

[63] Rust L Nizio KD Forbes SL The influence of ageing and surface type on the odour profile of blood-detection dog training aids. Anal Bional Chem. (2016) 61:6349–60. 10.1007/s00216-016-9748-9 27382970

[64] Agapiou Zorba E Mikedi K McGregor L Spiliopoulou C Statheropoulos M. Analysis of volatile organic compounds released from the decay of surrogate human models simulating victims of collapsed buildings by thermal desorption-comprehensive two-dimensional gas chromatography-time of flight mass spectrometry. Anal Chim Acta (2015) 883:99–108. 10.1016/j.aca.2015.04.024 26088782

[65] Perrault KA Nizio KD Forbes SL A comparison of one-dimensional and comprehensive two-dimensional gas chromatography for decomposition odour profiling using inter-year replicate field trials. Chromatographia (2015) 78:1057–70. 10.1007/s10337-015-2916-9

[66] Macias MS Harper RJ Furton KG A comparison of real versus simulated contraband VOCs for reliable detector dog training utilizing SPME-GC-M. Am Lab S (2008) 40:16–19.

[67] Rice S Koziel JA. Characterizing the smell of marijuana by odor impact of volatile compounds: an application of simultaneous chemical and sensory analysis. PLoS ONE (2015) 10:e0144160. 10.1371/journal.pone.0144160 26657499 PMC4684335

[68] Wiebelhaus N Hamblin D Kreitals NM Almirall JR Differentiation of marijuana headspace volatiles from other plants and hemp products using capillary microextraction of volatiles (CMV) couples to gas chromatography-mass spectrometry (GC-MS). Foren Chem. (2016) 2:1–8. 10.1016/j.forc.2016.08.004

[69] Kranz WD Strange NA Goodpaster JV “Fooling fido”- chemical and behavioral studies of pseudo-explosive canine training aids. Anal Bioanal Chem. (2014) 406:7817–25. 10.1007/s00216-014-8240-7 25424725

[70] Harper RJ Almirall JR Furton KG. Identification of dominant odor chemicals emanating from explosives for use in developing optimal training aid combinations and mimics for canine detection. Talanta (2005) 67:313–27. 10.1016/j.talanta.2005.05.019 18970171

[71] Prada PA Curran AM Furton KG Human Scent Evidence. Boca Raton, FL: CRC Press (2015).

[72] Waggoner LP Jones M Williams M Johnston JM Edge CC Petrousky JA Effects of extraneous odors on canine detection. Proc SPIE (1998) 3575:355–62.

[73] Prada PA Nevitt G Furton KG Testing the Ability of Birds to Detect Forensic Odorants: Comparison with Canine Abilities and Instruments. In: Proceedings of the American Academy of Forensic Sciences. Atlanta, GA: USA (2012).

